# Quality of Life Following Traumatic Brain Injury Among Older Adults

**DOI:** 10.1001/jamanetworkopen.2025.58087

**Published:** 2026-02-06

**Authors:** Kristen Apolinario, M. Muska Nataliansyah, Krista L. Haines, Patrick Murphy, Sarada Rao, Marc de Moya, Maddie R. Rundell, Amir N. Farah, Staci Young, Shelly D. Timmons, Rachel S. Morris

**Affiliations:** 1 Medical College of Wisconsin Medical School, Milwaukee; 2 Department of Surgery, Medical College of Wisconsin, Milwaukee; 3 Division of Trauma, Acute, and Critical Care Surgery, Department of Surgery, Duke University School of Medicine, Durham, North Carolina; 4 University of California San Francisco Fresno, Fresno; 5 Department of Family and Community Medicine, Medical College of Wisconsin, Milwaukee; 6 Department of Neurosurgery, Medical College of Wisconsin, Milwaukee

## Abstract

**Importance:**

The Glasgow Outcome Scale–Extended (GOS-E), Quality of Life After Brain Injury–Overall Scale (QOLIBRI-OS), and Patient Health Questionnaire–9 (PHQ-9) are commonly used to evaluate recovery after traumatic brain injury (TBI), but they may not fully reflect the lived experiences of older adults (≥65 years). As the population of older adult survivors of TBI grows, understanding the factors that shape recovery becomes increasingly important.

**Objective:**

To explore how older adults adapt to life in the first year after TBI and whether qualitative analysis of patient and caregiver interviews identifies aspects of recovery not captured by conventional outcome measures.

**Design, Setting, and Participants:**

This qualitative study used multiple-methods combining standardized surveys and semistructured interviews and was conducted from October 2023 to January 2025 at a level I trauma center in the US. Adults aged 65 years and older hospitalized for TBI were recruited through the institutional trauma registry and interviewed approximately 1 year after hospitalization. Patients with cognitive limitations could participate if a consenting caregiver proxy was available. Recruitment continued until theoretical saturation.

**Main Outcomes and Measures:**

The primary outcome was qualitative findings from thematic analysis of semistructured interviews. Secondary outcomes were health-related quality of life (QOLIBRI-OS); scale of 0 to 100 with higher scores indicating better perceived quality of life, functional status (GOS-E); scale of 8 levels with higher levels indicating good recovery, and depressive symptoms (PHQ-9); scale of 0 to 27 with higher scores indicating severe depression.

**Results:**

A total of 29 patients (12 [41.4%] female; mean [SD] age, 77.4 [7.2] years) and 13 caregivers participated in interviews and surveys, with 21 (73%) classified as having good recovery on GOS-E. Mean (SD) QOLIBRI-OS score was 70.1 (18.8), and median (IQR) PHQ-9 score was 4 (1-6). Three themes emerged from analysis of interviews: (1) influence of support systems on independence and quality of life; (2) adapting to postinjury life through grief, acceptance, and gratitude; and (3) desire for more information and guidance on life postinjury.

**Conclusions and Relevance:**

In this study, older adults and their caregivers reported that their post-TBI recovery was impacted by physical and emotional adaptation, caregiving dynamics, and understanding of their clinical course—factors not entirely reflected in standard outcome scales. These findings suggest that clear discharge counseling, structured caregiver education to maximize safety and independence, and regular follow-up could improve quality of life for older adults post-TBI.

## Key Points

**Question:**

Do quantitative survey measures of quality of life and functional status comprehensively assess recovery among older adults following traumatic brain injury?

**Findings:**

In this qualitative study using multiple methods with 29 patients and 13 caregivers, most participants scored in the good recovery range on quantitative measures. However, qualitative analysis of interviews revealed additional themes of adaptation, including reliance on support systems, emotional reframing, and the need for clearer guidance for life after discharge.

**Meaning:**

The findings of this study suggest that recovery after traumatic brain injury in older adults extends beyond what is reflected in standard scales, underscoring the importance of incorporating patient perspectives into outcome assessment.

## Teaser

This qualitative study investigates standard assessments used to evaluate recovery after traumatic brain injury and identifies aspects of recovery experienced by older adults that may not be captured by conventional methods.

## Introduction

Traumatic brain injury (TBI)–related hospitalizations in older adults (≥65 years) in the US have quadrupled over the past decade.^1,2^ This population experiences disproportionately poor outcomes due to age-related frailty, cerebral atrophy, and reduced neuroplasticity.^3,4^ After injury, they are more likely to experience prolonged hospitalizations, cognitive impairment, reduced mobility, and long-term disability, increasing their risk of institutionalization and loss of autonomy.^5,6,7,8^ Their recovery is further complicated by social and environmental factors, such as poor access to caregiving and geriatric rehabilitation services, low referral rates to postacute care programs, and financial constraints.^9,10,11^

The lack of understanding around how older adults adapt to life after TBI is a critical gap in trauma care.^12^ Prior research in this area has used traditional outcome measures, such as the Glasgow Outcome Scale (GOS) and the Quality of Life in Brain Injury (QOLIBRI) instrument. However, these metrics fall short in capturing the emotional, social, and psychological adjustments that substantially shape recovery experiences for older adults.^13,14^ QOLIBRI scores, for example, may indicate satisfaction even in the setting of functional decline due to response shift, in which individuals redefine their expectations or values after injury.^15^ In this context, QOLIBRI scores may indicate satisfaction even in the setting of objective decline.

To address this gap, we conducted a qualitative study using multiple methods (ie, standardized assessments and qualitative interviews) to explore how older adults adapt emotionally, socially, and functionally 1 year after injury. We hypothesized that thematic analysis of patient and caregiver interviews would identify domains of adaptation that extend beyond standard functional and quality-of-life scores. This approach aims to deepen understanding of how older adults adapt to life after injury, which could inform clinical decision-making, set realistic patient recovery goals, improve rehabilitation strategies, and ensure that long-term recovery efforts align with older adults’ needs and desires.^16^

## Methods

### Study Design and Data Collection

This qualitative study employed a multiple-methods approach, combining qualitative interviews and quantitative surveys to gain a comprehensive understanding of the experiences and recovery outcomes of older adults 1 year after TBI (either isolated or multiple trauma).^17^ The rationale for this approach was to capture both the quantifiable aspects of functional and psychological recovery and the nuanced, subjective experiences of adaptation and daily life following TBI. This approach also acknowledges that each method contributes distinct insights. All participants were identified through the institution’s level I trauma registry who met inclusion criteria were contacted. All surveys were performed after enrollment, at the time of interview, and with informed consent. This study was approved by the Medical College of Wisconsin Institutional Review Board (IRB). The IRB also granted a waiver of Health Insurance Portability and Accountability Act authorization requirements to review records for potential participants. Consolidated Criteria for Reporting Qualitative Research (COREQ) reporting guidelines were followed to enhance transparency in reporting. Reflexivity, team-based coding, and interviews with diverse respondents (ie, patients and caregivers) were employed to minimize bias and improve trustworthiness.

The inclusion criteria were older adults aged 65 years or older who sustained a TBI and were approximately 12 months postinjury. There was no upper age limit for the study. Exclusion criteria were non-English speaking participants or dead on arrival to the emergency department. To objectively characterize the functional and emotional recovery of participants, we employed 3 standardized survey instruments: the QOLIBRI–Overall Scale (QOLIBRI-OS), Patient Health Questionnaire–9 (PHQ-9), and the GOS–Extended (GOS-E).

### QOLIBRI-OS

The QOLIBRI-OS is a brief 6-item version of the complete QOLIBRI instrument, developed to measure health-related quality of life specifically for individuals with TBI. It addresses 6 domains: cognition, self-perception, daily life and autonomy, social relationships, emotions, and physical problems. Scores range from 0 to 100, with higher values indicating better perceived quality of life. The QOLIBRI-OS has been validated for use in large cohorts and correlates well with broader functional outcomes.^13,18^

### PHQ-9

The PHQ-9 is a validated 9-item screening tool for depressive symptoms, based on *Diagnostic and Statistical Manual of Mental Disorders* (Fourth Edition) criteria. It produces a total score from 0 to 27, with higher scores indicating more severe depression. A score of 0 to 4 indicates minimal depression; 5 to 9, mild depression; 10 to 14, moderate depression; 15 to 19, moderately severe depression; and 20 to 27, severe depression. The PHQ-9 has been utilized in post-TBI populations to assess depression severity.^19^

### GOS-E

The GOS-E is a commonly used functional outcome scale that classifies recovery into 8 levels: level 1, death; level 2, vegetative state; level 3, lower severe disability (SD); level 4, upper SD; level 5, lower moderate disability (MD); level 6, upper MD; level 7, lower good recovery (GR); level 8, upper GR. GR indicates return to independence with minimal or no deficits, MD reflects varying degrees of functional limitation despite independence, and SD is associated with ongoing dependence for daily activities. The GOS-E provides a more nuanced classification of functional status than the original 5-point Glasgow Outcome Scale, making it more sensitive to detecting moderate disability and partial recovery.^14,20^ These tools provided a quantitative framework for assessing functional outcomes, mood symptoms, and self-reported quality of life, which were analyzed alongside the qualitative narratives collected through interviews.

### Semistructured Interviews

The study team contacted patients and their caregivers via letter or phone. Individuals with severe cognitive impairments that impeded informed consent or interview participation were excluded, unless a consenting caregiver was available to serve as a proxy during interview. The goal was to recruit enough patients to achieve theoretical saturation.

The interviews were led by 2 trained faculty team members with prior experience in qualitative research. Interviewers asked questions regarding adaptation to disability, health-related quality of life, life before and after injury, and the overall recovery experience.

Demographic and clinical characteristics, including age, comorbidities, injury severity, length of stay, discharge destination, and postacute rehabilitation involvement, were abstracted from the medical record to provide clinical context. Because this study was exploratory and descriptive in design, these factors were summarized rather than analyzed as confounders or predictors, as the goal was to evaluate quantitative outcomes using experiential data rather than infer causality.

### Statistical Analysis

Interviews were audio-recorded, transcribed verbatim, and deidentified to ensure confidentiality. Transcripts were imported into Dedoose Version 9.2.012 (SocioCultural Research Consultants, LLC), a cloud-based qualitative data analysis platform that supports collaborative coding. The analytic team—faculty and trainees with experience in qualitative, trauma, or rehabilitation research—conducted thematic analysis primarily using a deductive coding approach.^21^ The initial codebook was based on the study’s interview domains: adaptation to disability, health-related quality of life, life before and after injury, and reflections on the overall recovery experience. The entire research team reviewed and approved the codebook before coding began.

At least 2 independent coders iteratively reviewed and applied codes to each transcript. The coding team met regularly to ensure consistency, resolve discrepancies, and group related codes into broader thematic categories reflecting patterns in participants’ experiences. Consensus discussions with the wider research team and documentation of coding decisions supported rigor, reliability, and reflexivity in the interpretation of participant narratives.^22^

Quantitative data from the QOLIBRI-OS, PHQ-9, and GOS-E were analyzed descriptively, and pairwise Spearman rank correlation coefficients were calculated to examine associations among quality of life, functional recovery, and mood symptoms. Statstical analyses were conducted with Microsoft Excel 2024 (Microsoft 365), statistical significance was set to *P* < 0.5. Spearman rank correlation was used to assess associations among outcome measures, given the nonparametric distribution of the data and the ordinal nature of the GOS-E scale.^23^ Descriptive statistics were generated to summarize the sample’s characteristics and recovery outcomes.

Quantitative and qualitative data were analyzed separately but in parallel, consistent with a multiple-methods design. The quantitative data (QOLIBRI-OS, PHQ-9, and GOS-E) were analyzed descriptively to characterize patient-reported quality of life, mood, and functional outcomes. The qualitative interviews provided complementary insights into the lived experiences behind these measures. During the interpretation stage, results across data types were cross-referenced to identify patterns and differences, contextualizing the quantitative findings rather than establishing direct statistical correspondence. This approach allowed for a more comprehensive understanding of adaptation after TBI, while acknowledging that statistical inference across data sources was not the goal.

## Results

### Quantitative Findings and Associations Among Primary Quantitative Measures

**Figure 1.**
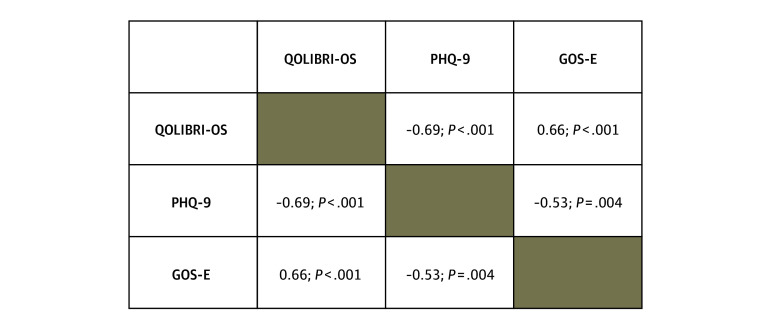
Spearman Rank Correlations Among Quality of Life After Brain Injury–Overall Scale (QOLIBRI-OS), Patient Health Questionnaire-9 (PHQ-9), and Glasgow Outcome Scale-Extended (GOS-E), With Data From 29 Participants

A total of 29 patients (12 [41.4%] female, 17 [58.6%] male; mean [SD] age, 77.4 [7.2] years) and 13 caregivers participated in recorded interviews with trained research staff (Table 1). Pairwise, Spearman rank correlation analyses revealed statistically significant associations among the main outcome measures (Figure 1). QOLIBRI-OS was negatively correlated with PHQ-9 (ρ = −0.69; *P* < .001) and positively correlated with GOS-E (ρ = 0.66; *P* < .001). PHQ-9 was also negatively correlated with GOS-E (ρ = −0.53; *P* = .004). These results were associated with higher functional recovery (GOS-E) as well as with better quality of life and fewer depressive symptoms. In contrast, greater depressive symptoms were associated with lower quality of life.

**Table 1.** Patient Characteristics

Characteristic	Participants, No. (%)	
	Male (n = 17)	Female (n = 12)
Age, mean (SD), y	76.0 (6.9)	76.3 (7.8)
Race^a^		
Asian	1 (5.9)	0
Non-Hispanic White	16 (94.1)	12 (100.0)
BMI, mean (SD)	28.8 (4.7)	28.0 (7.1)
Insurance status		
Medicare recipient	17 (100.0)	12 (100.0)
Social history		
Alcoholism	1 (5.9)	0
Smoking	8 (47.1)	4 (33.3)
Drug use	0	0
GCS score at presentation, median (range)	15 (7-15)	15 (14-15)
Injury Severity Score at presentation, median (range)	13 (4-26)	9.5 (1-50)
Initial computer tomography imaging findings		
Subdural hemorrhage	10 (58.8)	5 (41.7)
Subarachnoid hemorrhage	8 (47.1)	3 (25)
Cerebellar hemorrhage	0	1 (8.33)
Cerebral edema	3 (17.6)	1 (8.33)
Brain compression without herniation	1 (5.88)	1 (8.33)
Skull fracture	2 (11.8)	2 (16.7)
Concussion	0	2 (16.7)
Hospital length of stay, median (range), d	5 (1-38)	5 (1-63)
ICU length of stay, median (range), d	2 (0-11)	1 (0-6)
Discharge disposition		
Home	12 (70.6)	7 (58.3)
Subacute rehabilitation	2 (11.8)	3 (25.0)
Long-term acute care hospital	2 (11.8)	1 (8.3)
Skilled nursing facility	1 (5.9)	1 (8.3)
Comorbidities		
None	1 (5.9)	1 (8.3)
Hypertension	14 (82.4)	7 (58.3)
Diabetes	5 (29.4)	4 (33.3)
Obesity	1 (5.9)	1 (8.3)
Hyperlipidemia	6 (35.3)	6 (50.0)
Chronic obstructive pulmonary disease	2 (11.8)	1 (8.3)
Coronary artery disease	5 (29.4)	2 (16.7)
Chronic kidney disease	2 (11.8)	2 (16.7)
Depression	1 (5.9)	1 (8.3)
Seizures	0	1 (8.3)
Myocardial infarction	0	1 (8.3)
Atrial fibrillation	1 (5.9)	2 (16.7)
Congestive heart failure	2 (11.8)	1 (8.3)
Anticoagulant therapy	2 (11.8)	1 (8.3)
Cerebral vascular accident	1 (5.9)	0
Readmissions	2 (11.8)	1 (8.3)
Readmission reason		
Sinus arrest	1 (5.9)	0
Syncope and collapse	0	1 (8.3)
Surgery	0	1 (8.3)

Abbreviations: BMI, body mass index (calculated as weight in kilograms divided by height in meters squared); GCS, Glasgow Coma Scale; ICU, intensive care unit.

^a^ Patients self-identified their race, and those data are included here to fully characterize the demographics of the cohort.

#### QOLIBRI-OS Measure

**Figure 2.**
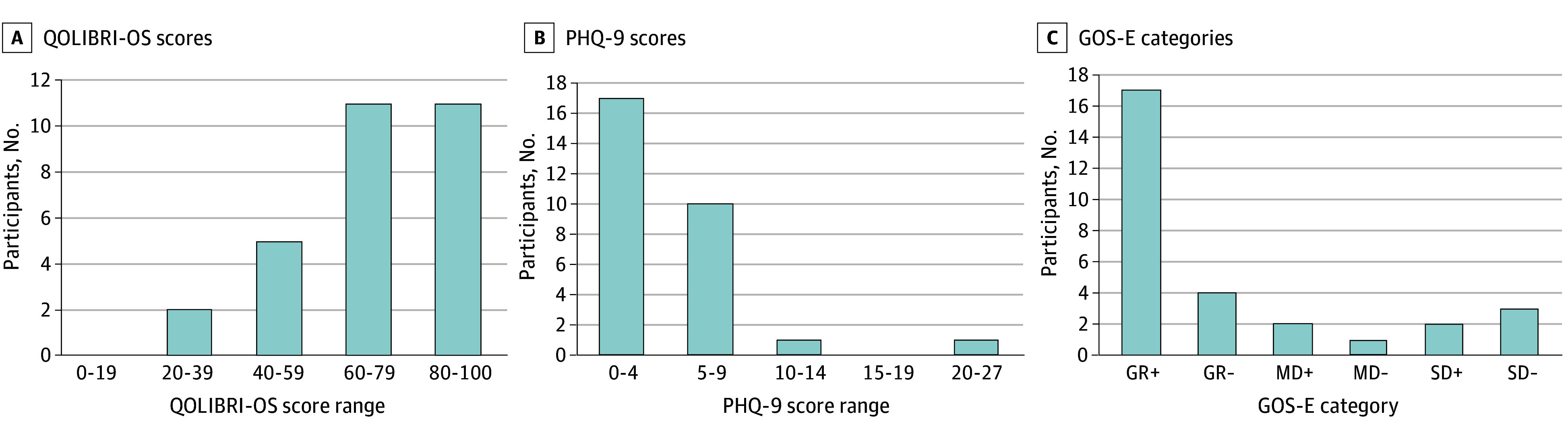
Distribution of Recovery Outcomes Among 29 Older Adults 1 Year After Traumatic Brain Injury A, Quality of Life after Brain Injury–Overall Scale (QOLIBRI-OS) score distribution. Higher scores indicate better health-related quality of life. B, Patient Health Questionnaire-9 (PHQ-9) score distribution. Higher scores indicate greater depressive symptom severity. C, Glasgow Outcome Scale–Extended (GOS-E) category distribution. Categories include good recovery (GR+, GR−), moderate disability (MD+, MD−), and severe disability (SD+, SD−); higher categories reflect greater functional independence.

Satisfaction with quality of life after TBI was measured using the QOLIBRI-OS (Figure 2A). Scores in this cohort ranged from 33.3 to 95.8, with a mean (SD) of 70.1 (18.8).

#### PHQ-9 Measure

Depressive symptoms were measured using the PHQ-9 (Figure 2B). Of those involved, 27 participants (93%) reported scores in the minimal to mild range, with a median (IQR) PHQ-9 score of 4 (1-6). Only 2 participants (7%) scored 10 or higher, consistent with moderate to severe depressive symptoms.

#### GOS-E Measure

Functional outcomes were assessed using the GOS-E (Figure 2C). Of those involved, 21 participants (73%) were classified as having upper or lower GR, 3 participants (10%) as upper or lower MD, and 5 participants (17%) as upper or lower SD.

### Qualitative Findings

Through iterative coding and thematic synthesis, 3 major themes emerged (Table 2). They were (1) the effect of support systems on independence and quality of life; (2) adapting to postinjury life through grief, acceptance, and gratitude; and (3) a desire for more information and guidance on life after TBI.

**Table 2.** Overview of Key Themes

Theme	Definition	Example quotation
Effect of support systems on independence and quality of life	Examines how family members and caregivers provide essential support for recovery, while also improving older adults’ sense of independence, self-identity, and the balance between receiving help and maintaining agency after TBI.	“On discharge ... my grandchildren, or granddaughters, were making stuff and bringing it over, and my kids were bringing food over. So, my support system is incredible … I’m very, very blessed that way with my family and being supportive.”
		“My sense of independence has been altered. … [My wife] doesn’t want me doing some things that I use do to by myself.”
Adapting to postinjury life through grief, acceptance, and gratitude	Describes the psychological process by which older adults come to terms with changes after TBI, moving through grief, adjusting expectations, and developing acceptance and gratitude as part of their emotional adaptation.	“I have ways of exercising, moving my neck around so that I can move it. And I’m happy. I’m happy to be alive.”
		“I’m just taking a day at a time. That’s what I tell people—as long as I wake up in the morning and I can be upright, I’m fine.”
Desire for more information and guidance on life postinjury	Highlights participants’ unmet needs for timely, comprehensive information and clear guidance following hospital discharge, and the importance of structured support to navigate the challenges of recovery and ongoing care.	“Nobody told me that I’d have issues with incontinence, which I did. And then I’m not sure anybody talked to me about how long it might be before I could actually sit up without pain.”
		“I wish that we had one person to sit down with us to say, your future care will involve a neurologist. Your future care will involve someone specifically for OT, for PT, for speech, and for cognition. We had no one person who was an overall person to guide us through this journey.”

Abbreviation: OT, occupational therapy; PT, physical therapy; TBI, traumatic brain injury

#### 1. Effect of Support Systems on Independence and Quality of Life

Codes related to social functioning, independence, adaptation strategies, effectiveness of adaptations, self-perception, preinjury activities, and postinjury difficulties were co-applied in these discussions. Assistance with mobility, household tasks, or emotional support provided a foundation for regaining quality of life. One participant reflected on the role the family had in providing tangible help and emotional grounding durinsg recovery:

On discharge ... my grandchildren, or granddaughters, were making stuff and bringing it over, and my kids were bringing food over. So, my support system is incredible. … I’m very, very blessed that way with my family and being supportive (female participant, in her 80s).

Patients grappled with a loss of independence that often contrasted with their preinjury routines. One participant reflected on this dynamic with his spouse: “My sense of independence has been altered. … [My wife] doesn’t want me doing some things that I used to do by myself” (male participant in his 60s).

Participants appreciated the support they received, noting that it was essential for their recovery. They also expressed a desire to retain as much independence as possible.

#### 2. Adapting to Postinjury Life Through Grief, Acceptance, and Gratitude

Another prominent theme was adaptation. This theme incorporated codes such as adaptation strategies, reflective insights, adjustment quality, cognitive function, mood, self-perception, and independence. These codes captured emotional and psychological techniques that participants used to cope with their lives after injury.

This process often involved grieving the loss of their prior abilities, followed by a gradual shift toward acceptance and, in many cases, a sense of gratitude for surviving the injury. As 1 participant shared:“I have ways of exercising, moving my neck around so that I can move it. And I’m happy. I’m happy to be alive” (female patient in her 80s).

Participants employed self-directed strategies, such as reframing limitations or focusing on what they could still enjoy, which facilitated emotional resilience and supported their day-to-day adaptation. As 1 participant described: “I’m just taking a day at a time. That’s what I tell people—as long as I wake up in the morning and I can be upright, I’m fine” (male patient in his 80s).

This exemplifies the perseverance shown by participants and their efforts to maintain quality of life despite functional limitations and uncertainty.

#### 3. Desire for More Information and Guidance on Life Postinjury

Despite these adaptive efforts, many participants reported feeling uncertain and isolated after discharge. Codes such as desired information, reflective insights, and initial information about life after injury frequently appeared in this domain. This information gap left patients and their families unprepared for challenges such as incontinence, pain, or delays in regaining function.

One patient was concerned about their physical condition after the injury:“Nobody told me that I’d have issues with incontinence, which I did. And then I’m not sure anybody talked to me about how long it might be before I could actually sit up without pain” (female patient in her 60s).

Others emphasized the need for clearer, more proactive communication from health care practitioners. A few suggested that having a dedicated point person or guide during the transition to outpatient care would have helped them navigate the complexity of follow-up appointments and therapies.

I wish that we had one person to sit down with us to say, your future care will involve a neurologist. Your future care will involve someone specifically for OT, for PT, for speech, and for cognition. We had no one person who was an overall person to guide us through this journey (male participant in his 60s).

Taken together, these accounts reveal gaps in postacute care that, if addressed, would better support older adults’ transitions from the hospital to home and promote a more coherent and informed recovery process.

## Discussion

This qualitative study used a multiple-methods exploratory approach, with separate quantitative and qualitative strands interpreted together to understand how measurable recovery outcomes and patient narratives intersect. Our results found that widely used outcome measures after TBI do not fully reflect the range of recovery experiences important to older adults.

Quantitatively, higher GOS-E scores correlated with higher QOLIBRI-OS scores and lower PHQ-9 scores, consistent with prior literature linking functional recovery, emotional well-being, and quality of life.^24,25,26^ However, participants’ descriptions of their recovery revealed aspects not captured by these measures. Older adults emphasized the importance of emotional adaptation, balancing support and autonomy, and the difficulties of navigating recovery without sufficient anticipatory guidance before discharge. These results suggest that improving quality of life after TBI in older adults involves more than just restoring function; it also requires fostering emotional resilience, identifying supportive caregiving networks, and ensuring patients are adequately prepared for recovery challenges.

Our study reinforces the notion that caregiving relationships are complex and profoundly influential in post-TBI adaptation.^27,28,29^ Although caregivers often provided substantial physical and emotional support, participants described tensions when support hindered independence or created unintended dependency. These findings align with previous research showing that caregiver involvement is most impactful when families are encouraged to promote patient autonomy, engage in collaborative goal-setting, and participate in shared decision-making, rather than assuming full responsibility for care.^30,31^ Approaches that support both caregivers and patients may improve the balance between safety, independence, and emotional well-being.

Posttraumatic growth and response shift,^15,32^ processes through which individuals recalibrate their identity and expectations after life-altering injury, were evident in participant interviews. Participants described grieving lost abilities, accepting new limitations, and expressing gratitude for aspects of life that remained possible. This reevaluation of meaningful physical and emotional function suggests that adaptation in long-term recovery involves redefining what constitutes a good quality of life after TBI, a continuous emotional process affected by both internal reflection and external support.

Our findings also highlight the persistent information gaps and lack of coordinated guidance experienced by older adults following TBI. Studies in trauma care have found that nurse navigators and multidisciplinary follow-up programs reduce readmissions, increase patient satisfaction, and improve functional outcomes by providing individualized education, proactive care coordination, and a consistent point of contact for patients and their families during recovery.^33,34,35^ Implementing such models in TBI care may help address the unmet needs identified in our cohort and support more successful long-term adaptation.

Given our findings, postdischarge interventions should consider incorporating structured caregiver assessments and training to help caregivers strike a balance between providing physical assistance and promoting patient independence and confidence in their abilities.^30,36^ Also, providing proactive education on navigating long-term symptoms and setting realistic recovery expectations could reduce uncertainty and improve transitions of care. Furthermore, ensuring implementation of TBI-specific care navigators can improve continuity of care and reduce patient and caregiver stress. Finally, behavioral health resources—such as counseling, peer support, and group therapy—should be integrated into follow-up care to help patients process their emotional responses. These strategies point toward a more holistic, patient- and caregiver-centered model of post-TBI care.

### Limitations

This study has several limitations. Its single-site design, limited racial and socioeconomic diversity, and purposive sampling strategy may restrict generalizability and fail to capture the full variability of TBI recovery experiences. Although clinical and sociodemographic factors were summarized to provide context, they were not modeled as predictors, consistent with the study’s exploratory purpose.

This study also did not include longitudinal follow-up data to track outcomes over multiple times points. Therefore, findings represent perceived adaptation and quality of life at one time point, rather than recovery trajectories or outcomes proportional to initial injury burden. Future research incorporating both clinical and patient-reported data across multiple time points could clarify how recovery expectations and lived experiences evolve with injury severity and rehabilitation.

While no direct statistical linking of datasets was performed, juxtaposing quantitative and qualitative perspectives offered a deeper understanding of recovery, highlighting that numeric outcomes alone may not fully reflect the extent of adaptation among older adults with TBI. Moreover, reliance on self-reported data might have introduced bias, particularly regarding participants’ emotional and social experiences. Additionally, our patients were generally less severely injured, as indicated by high Glasgow Coma Scale and GOS-E scores. Future research should involve multiple centers, diverse populations in terms of demographics and injury severity, and include caregiver proxies.

## Conclusions

The findings of this study suggest that recovery among older adults 1 year after TBI was shaped not only by functional status but also by emotional adaptation, caregiver dynamics, and the clarity of postdischarge guidance. Integrating qualitative insights with quantitative findings underscores the limitations of relying solely on traditional outcome metrics. Interventions that strengthen caregiver guidance, promote emotional resilience, provide clearer anticipatory information, and incorporate follow-up with care navigators may better support meaningful long-term recovery in this population.

## Notes

Supplement.
Data Sharing Statement
