# Supplementary material for: Quality of Life Following Traumatic Brain Injury Among Older Adults
Source: JAMA Netw Open. 2026 Feb 6;9(2):e2558087. doi: 10.1001/jamanetworkopen.2025.58087 (PMC12881979; doi:10.1001/jamanetworkopen.2025.58087)
Supplement: Supplement. — Data Sharing Statement [file jamanetwopen-e2558087-s001.pdf]

Data Sharing Statement

Apolinario. Quality of Life Following Traumatic Brain Injury Among Older Adults. *JAMA Netw Open*. Published February 06, 2026. doi:10.1001/jamanetworkopen.2025.58087

Data
Data available: No
